# Intestinal parasitic infection: prevalence, knowledge, attitude, and practices among schoolchildren in an urban area of Taiz city, Yemen

**DOI:** 10.3934/publichealth.2020059

**Published:** 2020-09-27

**Authors:** Talal Alharazi, Omar AA Bamaga, Nazeh Al-Abd, Jerold C Alcantara

**Affiliations:** 1 Department of Clinical Laboratory Sciences, College of Applied Medical Sciences, University of Hail, Hail, Kingdom of Saudi Arabia; 2 Department of Microbiology and Immunology, Faculty of Medicine and Health Sciences, Taiz University, Taiz, Yemen; 3 Department of Fundamental Medical Sciences, Faculty of Nursing, Hadhramout University, Hadramout, Yemen; 4 Department of Para-Clinic, Faculty of Medicine and Health Sciences, Aden University, Aden, Yemen

**Keywords:** intestinal parasitic infections, intestinal parasite, Taiz, Yemen, KAPs, schoolchildren

## Abstract

**Background:**

Intestinal parasitic infections (IPIs) are regarded as one of the main public health problems and socio-economic issues adversely affecting the health of millions of people worldwide. Our study aimed to describe the knowledge, attitude, and practices of local urban schoolchildren in Taiz City towards intestinal parasitic infections.

**Methods and material:**

This is a cross-sectional study conducted in Taiz, Yemen from March to May 2019. A total of 385 schoolchildren were selected using a random sampling technique from 7 primary schools. Wet-mount microscopic examination, formol-ether concentration techniques, and Lugols' iodine were employed in parasite detection and cyst identification.

**Results:**

Of the 385 schoolchildren examined for IPIs, 107 (27.8%) were positive for the presence of enteric parasites, some having multiple infections. The prevalence was slightly higher in males 46 (28.6%) than in females 61 (27.2%) but have no statistical difference (*P* = 0.77). *Entamoeba histolytica/dispar* was the most common infection with 16.4% of cases. A substantial percentage (40.5%) of the respondents displayed poor knowledge. The respondents also revealed inappropriate attitudes and practices that contribute to the prevalence of IPIs in the study.

**Conclusions:**

The study revealed the prevalence of intestinal parasites among the schoolchildren in Taiz, Yemen, suggesting that IPIs remain a major public health problem. *Entamoeba histolytica/dispar* was the most prevalent intestinal parasites identified among the schoolchildren. Age, poor knowledge of the mode of transmission, prevention, and acquisition of IPIs, and poor habitual hygiene practices increase the risk of acquiring intestinal infections.

## 1. Introduction

Intestinal parasitic infections (IPIs) are regarded as one of the main public health problems and socio-economic issues adversely affecting the health of millions of people worldwide particularly poor individuals in developing countries [1]. Frequently, IPIs are due to *Entamoeba histolytica, Giardia lamblia*, *Ascaris lumbricoides*, *Trichuris trichiura*, *Hookworms*, *Hymenolepis species*, *Taenia species* and *Schistosoma mansoni* where the majority of them are transmitted directly by fecal-oral route and others may be transmitted via skin penetration of humans [2].

IPIs are common cause of anemia such as iron, folate, and vitamin B12 deficiencies among low-income populations and are associated with micronutrient deficiencies such as low plasma vitamin A, loss of weight, diarrhea, chronic blood loss, and stunted growth among children [3]. Socio-demographic factors such as age, low-income, gender, family size, and education levels of parents, which have significantly associated with the increased prevalence of these parasites [4]. International studies showed that a significant percentage of IPIs were observed among schoolchildren. A prevalence rate of 19.6% in Zambia [5], in Khartoum, Sudan 30.0% [6], and in Riyadh, Saudi Arabia with 17.7% [7].

IPIs prevalence based on systematic review and meta-analysis study reported that *Giardia lamblia* as the main pathogenic agent in developing areas. However, a low prevalence of *Giardia lamblia* has been reported in South Asia (3%) and in Sub-Saharan Africa (2.7%), followed by the Middle East and North Africa [8],[9]. On the contrary, a high prevalence of *Giardia lamblia* and *Entamoeba histolytica* have been reported in different regions in Iran (8.5%, 5.7%) [10]. Several studies have also been conducted on intestinal parasites in many regions of Yemen such as Taiz City with 38.2%, Al-Mahweet governorate with 90.0%, and Sana'a with 54.8% prevalence rate [11]–[13].

In achieving successful and sustainable control program strategies, awareness, and involvement of the community are regarded as a significant instrument. Hence, evaluating an individual's knowledge, attitudes, and practices on intestinal infections in the community aids in recognizing, planning, and carrying out effective community-based intervention. To date, there is limited documented study on individuals' knowledge, attitude, and practices of intestinal parasitoids in Yemen. Our study aimed to describe the knowledge, attitude, and practices (KAPs) of local urban schoolchildren in Taiz city towards intestinal parasitic infection. These will serve as baseline data that will aid in the improvement and control of the disease.

## 2. Subjects and methods

### 2.1. Study area and ethical clearance

This cross-sectional school-based study was conducted in Taiz, Yemen from March to May 2019. The study was reviewed and approved by the Ethical Committee of the Faculty of Medicine and Health Sciences, Taiz University, Taiz, Yemen. Further, voluntary participation and consent from parents of the participants were obtained.

### 2.2. Study population and sample size

**Figure 1.**
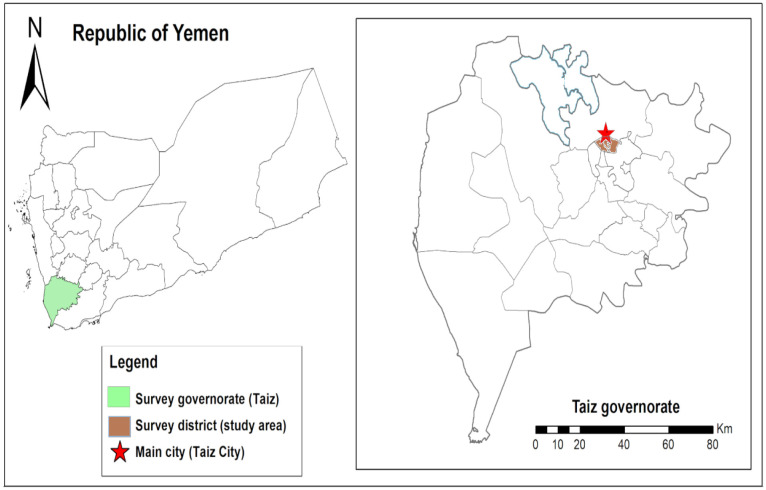
Map showing the study area on Yemen and Taiz governorate. The map was created using the Esri ArcGIS 10.7 software.

Seven (7) schools from the three administrative districts in Taiz city of Yemen participated and each district was represented by two (2) primary schools from Al-Qahiraa district and Al-Sala district, and three (3) from Al-Modafer district (Figure 1). From a list provided by each school, children from ages 7 to 15 years were randomly selected. The Epi Info statistical program was employed to determine the sample size. The hypothesized frequency of outcome factors in the population was 50%. Significance level was set at 0.05, design effect at 1, and 95% confidence interval resulting in a minimum sample size equal to 385.

### 2.3. Data collection and analysis of stool sample

A pre-tested interviewer-administered structured questionnaire was used to collect data, such as socio-demographic data. Students' knowledge, attitude, and practice were collected using a structured questionnaire by trained health workers.

For the collection of stool samples, the students were given a properly labeled wide-mouthed, sterile plastic vial with identifying marks, on the day of study. Appropriate ways of obtaining samples were carefully explained before the collection. Stool samples collected were transported and analyzed in the Parasitology Department at Taiz University. Laboratory techniques such as wet mount microscopic examination and formol-ether concentration techniques were employed in the detection of parasites while Lugols' iodine was used in the identification of cyst. Examination for the presence of ova, larvae, trophozoite, or cyst of intestinal parasites was carried out.

### 2.4. Statistical analysis

Data analysis was done using SPSS version 20 (SPSS Inc, Chicago, IL, USA). Categorical variables were presented using frequencies and percentages. Dependence between categorical characteristics was determined using cross-tabulations and Chi-square tests. *P*-values less than *0.05* were considered statistically significant.

## 3. Results

A total of 385 children were involved in this study. Of these 276 (71.7%), 71 (18.4%) and 42 (10.9%) were from Al Qahirah, Al Mudaffer, and Salh, respectively. Majority of the participants were aged 10 to 15 years old (79.2%) and predominantly females (58.2%). Slightly higher numbers (50.6%) of the participants were residents near sewage (Table 1).

Overall, a prevalence rate of 27.8% (107) for the presence of enteric parasites among the participants, with some having multiple infections. *Entamoeba histolytica/dispar* was noticed in 63 (16.4%), *Hymenolepis nana* in 15 (3.9%), *Giardia lamblia* in 8 (2.1%), *Ascaris lumbricoides* in 6 (1.6%), *Enterobius vermicularis* in 5 (1.3%), *Ancylostoma duodenale* in 4 (1%), *Trichuris trichiura* in 3 (0.8%) and *Schistosoma mansoni* in 3 (0.8%) of cases. *Entamoeba histolytica/dispar* was the most common infection recorded from the stool sample of participants and the overall intestinal parasite prevalence was slightly higher among males 46 (28.6%) than in females 61 (27.2%) than in but have no statistical difference (*P* = 0.77).

### 3.1. Knowledge about Intestinal Parasites (IP)

In general, a substantial percentage (40.5%) of the participants displayed poor knowledge about intestinal parasites. Noticeably, a high number of participants have reduced knowledge or unaware of the mode of transmission (65.7%), previous knowledge on IP (53.2%), symptoms of infection (47.3%), and prevention methods (44.4%). In addition, 37.9% of the participants were not aware if feces could be a source of infection. A large ratio (62.1%) of the participants were also previously infected (Table 2).

### 3.2. Attitude and practice about Intestinal Parasites

Notably, there were attitudes and practices of the respondents that pose risk to IPIs. In handwashing practices, substantial rates of the respondents were not using soap (43.4%). Further, 25.7% were washing their hands “sometimes” before eating, and a lower rate (1.6%) of the respondents was NOT washing their hands after defecation. A significant rate of inappropriate attitude and practices were also noticed including eating food outside the home (80.8%), wearing shoes “sometimes” when going outside (43.4%), swimming in places of water accumulation (39%), trimming or cutting nails “sometimes” (31.4%) and not always washing fruits or vegetables before eating (19.2%). Among the participants, 3.6% believed that playing around sewage cannot cause IP, and 4.4% of them were not aware if playing around sewage could lead to IP infection or not (Table 3).

**Table 1.** Sociodemographic characteristics of study respondents (N = 385).

Participants	Characteristics	Number	%
Gender	Male	161	41.8
	Female	224	58.2
Age	7–9	80	20.8
	10–15	305	79.2
Residence	Near to sewage	195	50.6
	Away from sewage	190	49.4

**Table 2.** Prevalence of intestinal parasites among Schoolchildren in the urban area of Taiz city based on sex.

Parasites species	Male No. (%)	Female No. (%)	Total No. (%)	*P* value
*Entamoeba histolytica/dispar*	24 (14.9%)	39 (17.4%)	63 (16.4%)	0.51
*Hymenolepis nana*	6 (3.7%)	9 (4.0%)	15 (3.9%)	0.88
*Giardia lamblia*	2 (1.2%)	6 (2.7%)	8 (2.1%)	0.33
*Ascaris lumbricoides*	3 (1.9%)	3 (1.3%)	6 (1.6%)	0.68
*Enterobius vermicularis*	3 (1.9%)	2 (0.9%)	5 (1.3%)	0.41
*Ancylostoma duodenale*	4 (2.5%)	0 (0.0%)	4 (1.0%)	0.01*
*Trichuris trichiura*	2 (1.2%)	1 (0.4%)	3 (0.8%)	0.38
*Schistosoma mansoni*	2 (1.2%)	1 (0.4%)	3 (0.8%)	0.38

Note:* Significant at level *p < 0.05*.

**Table 3.** Knowledge regarding intestinal parasites among the respondents (N = 385).

Question			Number	%
1	Previous knowledge on intestinal parasite?	Yes	180	46.8
		No	205	53.2
2	Have you ever been infected with IP?	Yes	251	65.2
		No	75	19.5
		Don't know	59	15.3
3	Feces as a source of infection?	Yes	239	62.1
		No	51	13.2
		Don't know	95	24.7
4	Symptoms of infection with IP	Know	203	52.7
		Don't know	182	47.3
5	Degree of harmfulness of sewage exposure	Harmful	363	94.3
		Harmless	3	0.8
		Don't know	19	4.9
6	Mode of transmission of IP	Know	132	34.3
		Don't know	253	65.7
7	Prevention methods of IP	Know	214	55.6
		Don't know	171	44.4
	Total knowledge	Good	229	59.5
		Poor	156	40.5

## 4. Discussion

This study was conducted to provide information on the prevalence of IPIs and their knowledge, attitudes, and practices. Incorporation of KAP surveys is recommended by the WHO as the keystone for health promotion campaigns. It is significant in helping programmers to adjust health education messages to improve public knowledge and attitudes regarding any public health concerns. Insufficient knowledge, attitude, and practices regarding intestinal parasites contribute to the high level of prevalence [14].

The overall prevalence of intestinal parasites in this study was 27.8%. The result differs from several studies in Yemen, in which the reported prevalence rate was considerably higher than our findings. In Haja town and in Taiz, a prevalence rate of 50.0% and 38.2% was reported, respectively, while a lower rate was found in Ibb with 7.4% [15]. On the regional level, the prevalence rate in this current study is markedly higher than in United Arab Emirates (7.7%) [16], Qatar (10.2%) [17], and in Iran (8.8%) [18]. The difference can be attributed to varied characteristics of the study population, differences in sample size, socio-economic, hygienic condition, geographical distribution, and diagnostic techniques employed to the participants.

The present study identified *Entamoeba histolytica/dispar* (33.6%) as the most prevalent intestinal parasite. Similar studies conducted in Nigeria recorded *Ascaris lumbricoides* as the common etiologic agent of intestinal parasites [19]. The high prevalence of *Entamoeba histolytica/dispar* in the current study could be possibly due to potable water contamination, poor handling of foodstuff, food contamination, and unhygienic practices such as not washing hands before eating meals or food.

Further, our study revealed that intestinal parasites were slightly higher among males (28.6%) than females (27.2%). Though, no statistically significant difference (*P* = 0.77) was found between IPIs and gender. Similar finding was found in the study conducted in Addis Ababa in which IPIs were more prevalent among males than females [20]. Conversely, the study of Amer et al. [14] in Saudi Arabia revealed that IPIs were higher among females. This difference may be due to the nature of the activities they do at home or outside, and their lifestyle.

Despite that majority of the participants in our study were familiar with IP, their knowledge of the symptoms, mode of transmission, and preventive measures were inadequate. Similar findings were reported in the study conducted in Malaysia [21]. Regarding the attitude and practice, greater numbers of respondents were knowledgeable and apply personal hygiene. Similar trends were found in the studies conducted in Abha and Egypt [22],[23]. In handwashing practices, the majority of the respondents in this study answered that they wash their hands before meals and after defecation. Comparatively, our finding was higher than the study conducted in Ethiopia [24] and Colombia [25]. The present study revealed that 25.7% and 1.6% of participants were “sometimes” washing their hands before eating and after defecation, respectively. The study conducted in Addis Ababa, Ethiopia showed that 17.1% and 14.4% do not practice hand washing before meals and after defecation, respectively [26]. Moreover, a significant number of respondents answered that washing fruits before eating, eating food outside the home, and cutting nails were being done “sometimes”. The lack of health information and general ignorance can contribute to poor knowledge and practices towards avoiding the source of IPIs among the participants in this study. In addition, laziness or calf out of play with friends or even the absence of facilities for handwashing near to latrines is considered bad hygiene practices. The present study shows a high number of respondents (96.0%) were using soap for hand washing. The study conducted in Abha [22] displayed fairly similar results in which more than 90.0% of the respondents use soap for handwashing. Noticeably, the current study also revealed that only 68.6% of the respondents cut their nails periodically. Findings in the study conducted in Saudi Arabia were slightly higher with 85% cutting their nails regularly [22].

## 5. Conclusion

Findings of the study revealed that a considerable ratio of school children in Taiz, Yemen was infected with IPIs. The prevalence of these intestinal parasites suggests that IPIs remains a major public health problem. Primarily, *Entamoeba histolytica/dispar* was the most prevalent intestinal parasites identified among the schoolchildren. Age, poor knowledge of the mode of transmission, prevention, and acquisition of intestinal parasite infections, and poor habitual hygiene practices increase the risk of acquiring intestinal infections. Enhancing the awareness of intestinal parasites among the schoolchildren and their parents through health education campaigns, and consistent and proper guidance on good personal hygiene practices are recommended.
